# Mitogenomic Characterization of Cameroonian Endemic *Coptodon camerunensis* (Cichliformes: Cichlidae) and Matrilineal Phylogeny of Old-World Cichlids

**DOI:** 10.3390/genes14081591

**Published:** 2023-08-06

**Authors:** Shantanu Kundu, Piyumi S. De Alwis, Ah Ran Kim, Soo Rin Lee, Hye-Eun Kang, Yunji Go, Fantong Zealous Gietbong, Arif Wibowo, Hyun-Woo Kim

**Affiliations:** 1 Department of Marine Biology, Pukyong National University, Busan 48513, Republic of Korea; shantanu1984@gmail.com (S.K.); piumisandaruwanidealwis@gmail.com (P.S.D.A.); 2 Marine Integrated Biomedical Technology Center, National Key Research Institutes in Universities, Pukyong National University, Busan 48513, Republic of Korea; ahrankim@pukyong.ac.kr (A.R.K.); srlee090@pukyong.ac.kr (S.R.L.); 3 Institute of Marine Life Science, Pukyong National University, Busan 48513, Republic of Korea; kanghe24@pukyong.ac.kr; 4 Industry 4.0 Convergence Bionics Engineering, Pukyong National University, Busan 48513, Republic of Korea; ligal82@naver.com; 5 The Ministry of Livestock, Fisheries and Animal Industries (MINEPIA), Yaoundé 00237, Cameroon; f_giet@yahoo.com; 6 Research Center for Conservation of Marine and Inland Water Resources, National Research and Innovation Agency (BRIN), South Tangerang 15314, Indonesia; wibarf@yahoo.com

**Keywords:** African cichlids, mitochondrial genome, cladistics, geological history, rift system, endemic radiation

## Abstract

The mitogenomic evolution of old-world cichlids is still largely incomplete in Western Africa. In this present study, the complete mitogenome of the Cameroon endemic cichlid, *Coptodon camerunensis,* was determined by next-generation sequencing. The mitogenome was 16,557 bp long and encoded with 37 genes (13 protein-coding genes, two ribosomal RNA genes, 22 transfer RNA genes, and a control region). The *C. camerunensis* mitogenome is AT-biased (52.63%), as exhibited in its congener, *Coptodon zillii* (52.76% and 53.04%). The majority of PCGs start with an ATG initiation codon, except COI, which starts with a GTG codon and five PCGs and ends with the TAA termination codon and except seven PCGs with an incomplete termination codon. In *C. camerunensis* mitogenome, most tRNAs showed classical cloverleaf secondary structures, except tRNA-serine with a lack of DHU stem. Comparative analyses of the conserved blocks of two Coptodonini species control regions revealed that the CSB-II block was longer than other blocks and contained highly variable sites. Using 13 concatenated PCGs, the mitogenome-based Bayesian phylogeny easily distinguished all the examined old-world cichlids. Except for Oreochromini and Coptodinini tribe members, the majority of the taxa exhibited monophyletic clustering within their respective lineages. *C. camerunensis* clustered closely with *Heterotilapia buttikoferi* (tribe Heterotilapiini) and had paraphyletic clustering with its congener, *C. zillii*. The Oreochromini species also displayed paraphyletic grouping, and the genus *Oreochromis* showed a close relationship with Coptodinini and Heterotilapiini species. In addition, illustrating the known distribution patterns of old-world cichlids, the present study is congruent with the previous hypothesis and proclaims that prehistoric geological evolution plays a key role in the hydroclimate of the African continent during Mesozoic, which simultaneously disperses and/or colonizes cichlids in different ichthyological provinces and Rift Lake systems in Africa. The present study suggests that further mitogenomes of cichlid species are required, especially from western Africa, to understand their unique evolution and adaptation.

## 1. Introduction

The family Cichlidae (order Cichliformes) is one of the most species-rich groups, with 1750 recognized species classified under 252 genera [1]. They are found in a wide range of aquatic environments throughout India, Africa, and South and Central America, including lakes and riverine systems. Cichlids are important food fish that are commonly employed in aquaculture avenues, as well as popular aquarium fish with high decorative values [2]. This teleost lineage also provides an exciting chance to investigate its biological characteristics in a variety of ways due to its astonishing diversity. In a recent review article, many aspects of cichlid fish biology were discussed, with a focus on East African rift lake cichlids [3]. Cichlids have been frequently developed by a combination of unusual phenotypic traits (such as color variation, fin forms, brain size, sex, etc.) and distinctive behavioral traits [4,5,6,7,8,9]. The reproductive isolation and speciation of cichlids, however, have also been attributed to their distinct reproductive behavior [10]. In addition, ancient hybridization and following phenotypic uniqueness have stimulated cichlid fish radiation in African Lake systems [11,12,13,14]. 

In the Neotropical region, multi-locus phylogeny and exon-based phylogenomic approaches have been employed to resolve the taxonomic conundrum, systematics position of competing clades, and evolutionary trend [15,16,17,18]. Furthermore, genomic data are being used to forecast continental radiations with the adaption and speciation of cichlid fishes in the Neotropics [19,20,21]. Although the variety of cichlid fishes has been repeatedly examined in the Afrotropical area, researchers are still whispering about their adaptability and development, particularly in East African and West African rift systems in relation to prehistoric biogeography [22,23,24,25,26]. This restricted study, however, was designed to demonstrate the diversity and phylogeny of cichlids from the Asian continent [27]. Simultaneously, the investigation of cichlids and species discovery is currently underway by merging genomic information with classical taxonomy [28,29]. The information included in DNA sequences might also be used to pretend that biological variables such as selective mating, neutral polymorphisms, and sympatric speciation influences the speciation of cichlid fish on the African continent and their distinctive foraging adaption [30,31]. Nonetheless, ecological processes and environmental changes pressurized the African hydroclimate, greatly activating the speciation of cichlid fishes in various riverine systems and lakes [32,33,34,35].

Molecular data were to determine the population structure of cichlid fish from different river basins and East African lakes [36,37,38,39]. Many large-scale genomic initiatives using phylogenomic techniques have been undertaken to shed light on the phylogenetic connection of the major African cichlid lineages, as well as their gene flow and diversification [40,41,42,43]. In addition, nuclear and mitochondrial markers targeting distinct taxonomic lineages were developed to elucidate their evolutionary relationships [44,45,46,47,48,49,50,51]. Molecular data were also used to assess the divergence time of several cichlid lineages in connection with fossil records and the geological time scale [52,53]. Later, complete mitochondrial genome investigations were planned to reveal the phylogenetic connection of numerous cichlid fishes [54,55]. Because of its large genome size, conserved structure, and fast evolutionary rates, mitogenomic data are a useful tool for examining the biodiversity, phylogeny, and evolutionary connections of species across a wide variety of taxa [56,57]. The utility of complete mitogenome sequences has been verified for understanding evolutionary connections, including population genetics and the conservation status of fish species [58,59,60]. Prior to this study, 67 old-world cichlids species of mitogenomes were generated from Africa, which often resulted in a biased perspective of the evolution of many diverse lineages.

The genus *Coptodon* includes 32 valid species that are endemic to Africa and the Middle East [1]. This group of fish, known as tilapia, belongs to the haplotilapiine lineage and has a long-lasting dilemma in their classification. Initially, the systematic review of *Coptodon* was carried out based on morphological characteristics, including dentition, body shape, coloration, and meristic and morphometric traits [61]. Later, molecular data were used to examine the monophyly, origin, and diversification of *Coptodon* [62]. The integration of molecular techniques altered *Coptodon* species classification and placed them in the newly defined tribe ‘Coptodonini’ [63,64]. The genetic diversity, population genetic structure, and hidden cryptic diversity of *Coptodon* species were recognized in Africa by the rapid success of molecular methods [65,66,67,68]. Despite their high species diversity, the mitogenomic library of *Coptodon* species is sparse in the worldwide GenBank database, with two mitogenomic sequences of a single species, *Coptodon zillii* (KM658974 and MW194077), which were produced to understand mitogenomic characteristics and phylogeny [69,70]. Thus, the current study aims to generate the first full mitogenome of *Coptodon camerunensis* from Cameroon in order to address the gap in mitogenomic-based phylogenetic evaluations. This species is endemic to Cameroon and is classified as ‘Vulnerable’ by the International Union for Conservation of Nature’s (IUCN) Red List of Threatened Species [71]. We anticipate that the current mitogenomic information will increase evolutionary knowledge of this group of cichlids, which are predominantly found in western Africa because genetic data are critical for understanding the biology and conservation needs of endemic and vulnerable species [72]. The structure and variations in *C. camerunensis* mitochondrial genes, as well as their comparison with the *C. zillii* mitogenome, can contribute to our understanding of genetic evolution. The large-sized DNA sequences of the targeted species will aid in estimating the genetic diversity and population structure of this unusual cichlid fish species in the near future. Furthermore, continental drift and subsequent geological processes, such as drainage evolution, disseminate and colonize fish variety, including cichlids, are also explored throughout African continents [73,74,75]. The current study further intends to unravel the matrilineal phylogeny of old-world cichlids and explore their radiations in various ecosystems that may have been induced by geological processes. More genetic information on all extant lineages, the estimation of divergence time, with geographic distribution modeling can all help to improve our understanding of the origin and diversification of old-world cichlids.

## 2. Materials and Methods

### 2.1. Sampling and Species Identification

**Figure 1:**
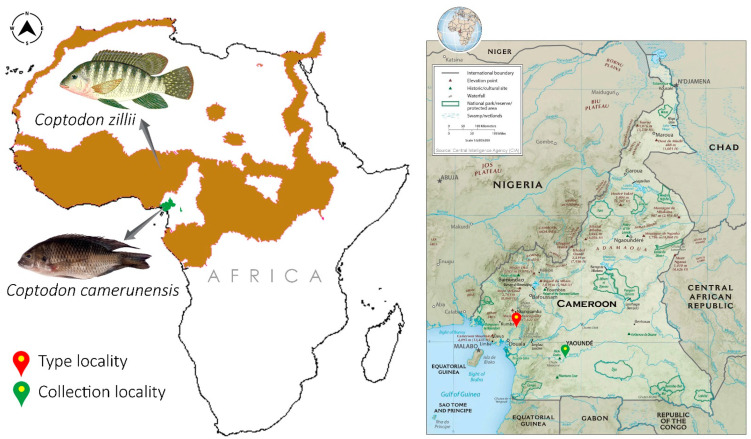
Map showing the IUCN range distribution pattern of two Coptodonini species (*C. camerunensis* and *C. zillii*) in Africa. The red and green pin show the type locality and collection locality of *C. camerunensis*, respectively.

The *Coptodon* species specimen was procured from the Nyong River in Cameroon (3.765 N 12.245 E) (Figure 1). The Institutional Animal Care and Use Committee (Approval Code: PKNUIACUC-2022-72 dated 16 December 2022) of the host institute authorized the use of muscle tissue from deceased animals. The specimen was tentatively identified as *C. camerunensis* based on physical characteristics [76]. A sufficient amount of tissue was extracted from the organism and kept at 4 °C in a 2 mL centrifuge tube with 70% ethanol. At Pukyong National University in South Korea’s Department of Marine Biology, samples of muscle tissue and genomic DNA were collected. The entire specimen was vouchered at the Ministry of Livestock, Fisheries and Animal Industries (MINEPIA), Yaoundé. Using known primers (FISH-BCL and FISH-BCH), we amplified incomplete mitochondrial COI sequences to confirm species identification [77]. The synthesized sequences exhibited a 99% identity to the *C. camerunensis* published sequence (KJ938224) [78]. The range of distributions (.shp files) for *C. camerunensis* and *C. zillii* were acquired from the IUCN (https://www.iucnredlist.org/ (accessed on 15 July 2023) and mapped using ArcGIS 10.6 software (ESRI1, CA, USA).

### 2.2. DNA Extraction, Mitogenome Sequencing, and Assembly

The genomic DNA were extracted using the AccuPrep^®^ Genomic DNA extraction kit (Bioneer, Daejeon, Republic of Korea) and a conventional procedure. The quality and quantity of gDNA were examined through a NanoDrop spectrophotometer (Thermo Fisher Scientific D1000, Waltham, MA, USA). The TruSeq Nano DNA High Throughput Library Prep Kit (Illumina) manufacturer’s instructions were followed in the preparation of the sequencing libraries. Briefly, 100ng of genomic DNA was trimmed using adaptive focused acoustic technology (Covaris), and the unplugged DNA was end-repaired to make 5′-phosphorylated, blunt-ended dsDNA molecules. The TruSeq DNA UD Indexing adapters were ligated to these DNA fragments after a single ‘A’ nucleotide was added. To construct the final DNA library, the products were cleaned and enriched by PCR. The libraries were quantified using qPCR and certified using the Agilent Technologies 4200 TapeStation D1000 screentape (Agilent Technologies). Paired-end (2 × 150 bp) sequencing was executed using the NovaSeq (Illumina) available at Macrogen (https://dna.macrogen.com/), Daejeon, Republic of Korea. High-quality paired-end reads were assembled by the Geneious Prime version 2023.0.1. Further, to obtain the full length of the control region, species-specific primers (5′-GCAACGAGGATTGACGTTCC-3′ and 5′-GGCTAAGCAAGGTGTTATGG-3′) were designed in the present study. The PCR was carried out by a TaKaRa verity thermal cycler containing a 1X PCR buffer, 1 U Taq polymerase, 10 pmol primers, 2.5 mM dNTPs, and 1 µL template DNA. The purification of the PCR products was performed by an AccuPrep^®^ PCR/Gel purification kit (Bioneer, Republic of Korea). The amplicons were amplified with the BigDye^®^ Terminator v3.1 Cycle Sequencing Kit (Applied Biosystems) and sequenced bi-directionally using the ABI PRISM 3730XL DNA analyzer housed at Macrogen (https://dna.macrogen.com/), Republic of Korea. The control region was assembled with the complete mitogenome by assuring the overlying regions’ alignment through MEGA X after eliminating the noisy parts through SeqScanner version 1.0 (Applied Biosystems Inc., CA, USA). The direction and boundary of each gene were confirmed through MITOS v806 (http://mitos.bioinf.uni-leipzig.de, accessed on 15 July 2023) and MitoAnnotator (http://mitofish.aori.u-tokyo.ac.jp/annotation/input/, accessed on 15 July 2023) web servers [79,80]. The boundaries of the protein-coding genes (PCGs) were further assured after checking the putative amino acids sequence array of vertebrate mitochondrial genes. The generated mitogenomes were submitted to the GenBank global database through the Bankit submission tool (https://www.ncbi.nlm.nih.gov/WebSub/, accessed on 15 July 2023) along with the standard five-column feature table. 

### 2.3. Genomic Characterization 

The spherical view of the *C. camerunensis* mitogenome was designed through a MitoAnnotator (http://mitofish.aori.u-tokyo.ac.jp/annotation/input/, accessed on 15 July 2023). The mitogenomic structure and variations were compared with the two available mitogenomes of a single congener, *C. zillii* (MW194077, generated from the known range distribution, and KM658974, generated from Borabu dam, Lake Victoria basin, Kenya). We calculated the overlapping regions and the intergenic spacers between the neighboring genes manually. The nucleotide compositions of PCGs, ribosomal RNA (rRNA), transfer RNA (tRNA), and the control region (CR) were estimated using MEGA X [81]. The base composition skews were also calculated by following the previous research: AT-skew = [A − T]/[A + T], GC-skew = [G − C]/[G + C] [82]. The start and stop codons of each PCG were affirmed through MEGA X based on the vertebrate mitochondrial genetic code. The gene boundaries of rRNA and tRNA genes were also validated through tRNAscan-SE Search Server 2.0 as well as ARWEN 1.2 [83,84]. The structural domains of CR were identified through CLUSTAL X alignments [85], and tandem repeats were examined by the online Tandem Repeats Finder web tool (https://tandem.bu.edu/trf/trf.html, accessed on 15 July 2023) [86]. 

### 2.4. Phylogenetic Analyses

To understand the matrilineal phylogenetic relationships, all accessible African Cichlids mitogenomes were obtained from the GenBank database (Accessed on 19 April 2023) (Table S1). The mitogenome of *Mugil cephalus* (AP002930) (family Mugilidae) was incorporated into the dataset as an outgroup. All 13 PCGs were concatenated using the iTaxoTools 0.1 tool to build the dataset for phylogenetic analysis [87]. The best fit ‘GTR+G+I’ model was estimated with the lowest BIC value in MEGA X. The Metropolis-coupled Markov chain Monte Carlos (MCMCs) method was used by Mr. Bayes 3.1.2 to construct the Bayesian (BA) tree. The MCMCs were run for 10,000,000 generations, with tree sampling occurring every 100 generations, with 25% of the samples discarded due to burn-in [88]. The constructed topology was further illustrated by the iTOL v4 webserver (https://itol.embl.de/login.cgi, accessed on 15 July 2023) [89].

## 3. Results and Discussion 

### 3.1. Mitogenome Structure and Organization 

**Figure 2:**
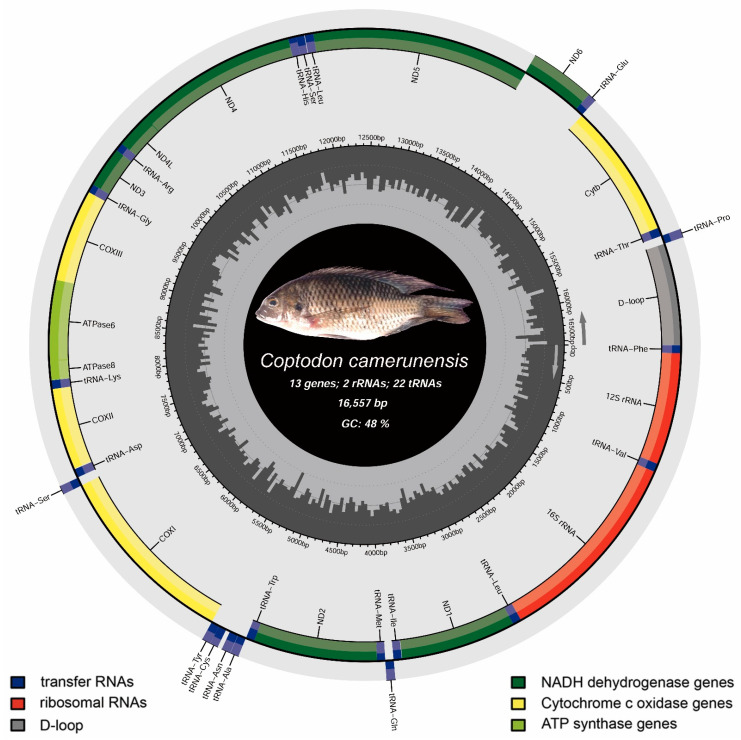
Mitochondrial genome of *C. camerunensis* drawn by the MitoAnnotator online server.

In the present study, *C. camerunensis* mitogenome (16,557 bp) was determined (GenBank accession number OQ696044) for the first time. A total of 13 protein-coding genes (PCGs), 22 transfer RNA genes (tRNAs), 2 ribosomal RNA genes (rRNAs), and a non-coding AT-rich regulatory region (CR) were found in the circular mitogenome of *C. camerunensis*. The heavy strand had 28 genes (12 PCGs, 2 rRNAs, and 14 tRNAs), whereas the light strand contained NAD6 and eight tRNAs (trnQ, trnA, trnN, trnC, trnY, trnS2, trnE, and trnP) (Table 1, Figure 2). The *C. camerunensis* mitogenome had six overlapping sections totaling 92 bp in length, with the longest overlapping region (69 bp) found between the tRNA-Pro (P) and control regions. *C. zillii* (KM658974) also showed six overlapping regions (24), while the other mitogenome (MW194077) showed seven overlapping regions (25), with the longest overlapping region (10) detected between ATP synthase 8 (atp8) and ATP synthase 6 (atp6) genes. Furthermore, *C. camerunensis* included 10 intergenic spacer regions totaling 58 bp in length, with the longest stretch (35 bp) located between trnN and trnC. *C. zillii* mitogenome (KM658974) had nine intergenic spacer regions with a total length of 55 bp, whereas the other mitogenome (MW194077) was marked with the highest (11with 59 bp) among the three *Coptodon* species (Table S2).

The *C. camerunensis* mitogenome was AT-biased (52.63%), with 27.12% A, 16.38% G, 30.99% C, and 25.51% T. A Similar AT richness was detected in the nucleotide composition of the other two *Coptodon* mitogenomes ranging from 52.76% (*C. zillii*, MW194077) to 53.04% (*C. zillii*, KM658974) (Table 2). The mitogenome of *C. camerunensis* was recorded with 0.031 and −0.308 AT skew and GC skew, respectively. A comparative study of the other two *Coptodon* mitogenomes revealed that the AT skew varied from 0.027 to 0.028, and the GC skew ranged from 0.299 to 0.306 (Table 2). A similar pattern of the nucleotide composition and AT biases were reported in previously characterized fish mitogenomes [90,91]. The genetic diversity detected in *Coptodon* mitogenomes could be related to their evolutionary process and energy metabolism, as demonstrated in other fish [92]. The work shed insight on numerous structural traits of *Coptodon* mitogenomes, and such pragmatic data are crucial for determining the roles of the mitogenomes and encoding genes.

### 3.2. Protein-Coding Genes 

In *C. camerunensis*, 13 PCGs accounted for 69.29% of the mitogenome, measuring 11,472 bp. The shortest PCG was ATP8, with a length of 168 bp, while the largest was NAD5, with a length of 1839 bp. Furthermore, *C. zillii* (KM658974) and *C. zillii* (MW194077) both marked the same size as the *C. camerunensis* with 11,472 bp for a total of 13 PCGs. The total PCGs of *C. camerunensis* were AT-biased (51.59%), whereas the AT skew and GC skew were −0.067 and −0.332, respectively. The majority of the *C. camerunensis* PCGs began with an ATG (Methionine) initiation codon, whereas COI began with a GTG (valine) codon (Table 2). A similar pattern of initiation codons was also found for all PCGs from the other two *Coptodon* species. The conventional TAA codon was found among five PCGs (NAD1, COI, ATP8, NAD4L, and NAD5), but the incomplete stop codons (TA-) and (T--) were found among NAD2 and ATP6, and among COII, COIII, NAD3, NAD4, and CYTB, respectively. Moreover, a unique feature was observed in *C. camerunensis* ND6 PCG, which was terminated by the TAG stop codon, while in other mitogenomes of *C. zillii*, this gene was terminated by the TAA stop codon. The termination codon for most of the PCGs was the same in *Coptodon* mitogenomes; however, the COI of *C. zillii* (KM658974) was terminated by the TAG stop codon, and the *C. zillii* mitogenome (MW194077 and KM658974) was detected with the TAG codon as a termination codon for NAD1 gene (Table S3). By adding a poly-A tail during RNA processing, these incomplete stop codons could be terminated with TAA [93]. As with other fish species, the identified genetic differences might result in the independent selection of PCGs [94,95]. The PCGs perform critical functions in oxidative phosphorylation and ATP synthesis and encode proteins in the electron transport pathways. As a result, the addition of mitogenomes from various *Coptodon* species can be evaluated to find variations in gene expression and energy use.

### 3.3. Ribosomal RNA and Transfer RNA Genes

**Figure 3:**
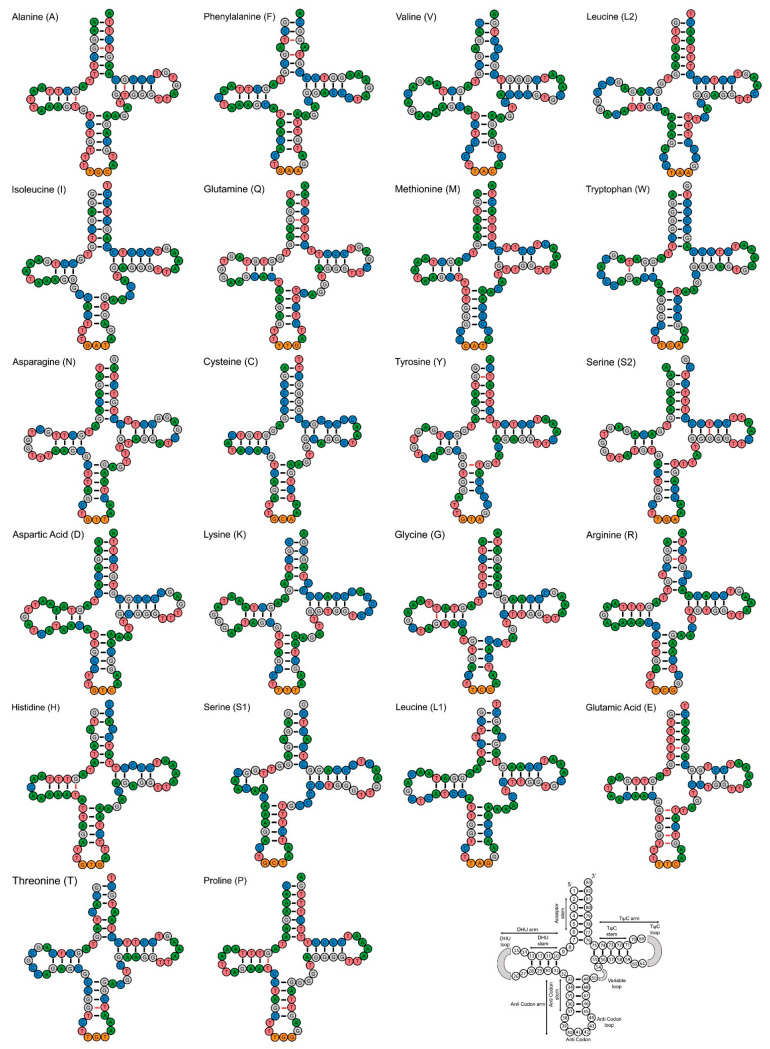
Secondary structures of 22 transfer RNAs (tRNAs) of *C. camerunensis* showing structural diversity. Full names and IUPAC-IUB single letter amino acid codes are used to identify the tRNAs. The final structure depicts the nucleotide locations and features of the stem-loop of tRNAs. Black and red color bars represent the Watson–Crick and wobbling base pairings, respectively.

The ribosomal RNA genes in *C. camerunensis* were 2636 bp long (15.92% of the total mitogenome), comprised a short ribosomal RNA (12S rRNA) and a big ribosomal RNA (16S rRNA), with lengths of 945 bp and 1691 bp, respectively. The length of the rRNAs of *C. camerunensis* was the lowest when compared to the other two mitogenomes: 2637 bp in *C. zillii* (KM658974) and 2638 bp in *C. zillii* (MW194077) (Table 2). AT richness in ribosomal RNA varied between 52.90% (*C. zillii*, KM658974) and 53.14% (*C. zillii*, MW194077). The AT skew in the ribosomal RNA varied from 0.210 (*C. zillii*, KM658974) to 0.212 (*C. camerunensis*), while the GC skew ranged from 0.126 (*C. zillii*, KM658974) to 0.123 (*C. camerunensis*) (Table 2). The structure and variations in rRNA genes, particularly for the highly conserved loops, provided significant insight into the catalytic chemical reactions that took place during the synthesis of the proteins [96]. Furthermore, the *C. camerunensis* mitogenome consisted of 22 tRNA genes whose length ranged from 66 bp (trnC) to 74 bp (trnK), with a total length of 1554 bp (9.39% of the total mitogenome), which was similar to that recorded for *C. zillii* (MW194077). Moreover, the lowest complete length (1553 bp) for transfer RNA Genes was recognized in *C. zillii* (KM658974). The 22 tRNA genes of all three *Coptodon* mitogenomes were AT-biased, ranging from 54.7% (*C. camerunensis*) to 54.93% (*C. zillii*, KM658974). The range of AT skew was 0.020 (*C. zillii*, KM658974) to 0.031 (*C. camerunensis*), and GC skew was 0.037 (*C. camerunensis* and *C. zillii*, MW194077) to 0.049 (*C. zillii*, KM658974) (Table 2). Except for trnS1, which lacked the DHU stem, most of the tRNAs were predicted to fold into the typical cloverleaf secondary shape [97,98]. These genetic features are essential for the formation of secondary RNA structures and their function in a range of biological systems [99]. According to the comparative structural features of tRNAs, 13 tRNA genes (trnA, trnF, trnL2, trnQ, trnW, trnY, trnS2, trnG, trnR, trnH, trnE, trnT, and trnP) were constituted by both conventional Watson–Crick base (A=T and G≡C) pairing and wobble base pairing (G-T) while the remaining nine tRNA genes were only built with Watson–Crick base pairs (Figure 3).

### 3.4. Features of Control Region 

**Figure 4:**
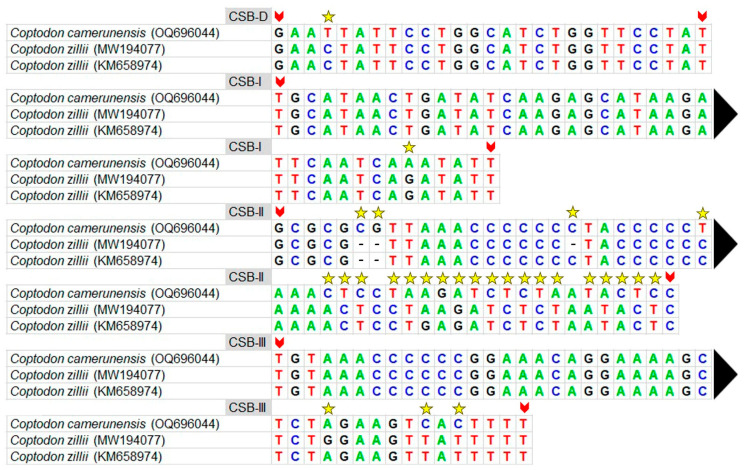
Control regions of two *Coptodon* species with structural variations in the four conserved domains. The beginning and ending positions of the conserved domains are shown by red arrows. The variable regions are marked with yellow stars.

The overall length of *C. camerunensis* CR was 929 bp, with a 62.86% AT and 37.14% GC content. The full-length sequence of the CR was recovered from two separate mitogenomes of the *C. zillii* species at two different locations, spanning from 852 bp (MW194077) to 924 bp (KM658974). The AT skew ranged from −0.020 (*C. zillii*, KM658974) to 0.027 (*C. camerunensis*), and GC skew was −0.212 (*Coptodon camerunensis*) to −0.142 (*Coptodon zillii*, MW194077) (Table 2). Neither *C. camerunensis* nor *C*. *zillii* mitogenomes contained tandem repeats. The conserved block locations (CSBD, CSBI, CSBII, and CSBIII) were determined to be comparable in both *C. camerunensis* and *C. zillii*, as documented in other teleost fishes [90,91,97]. The length of the CSBII block was the longest (52 bp) when compared to the other blocks, which were CSBD (27 bp), CSB-I (41 bp), and CSBIII (43 bp). In CSB-II, the comparison analysis revealed highly variable nucleotide sites and parsimony-informative nucleotides (Figure 4). This AT-rich regulatory region could be utilized to assess the population structure of any species. It was possible to identify the inter- and intra-specific differences among *Coptodon* species using these variable nucleotides. As demonstrated in other species, similar studies on conserved domains may be required to understand the replication and transcription of the mitochondrial genome [90,91].

### 3.5. Mitogenomic Phylogeny 

**Figure 5:**
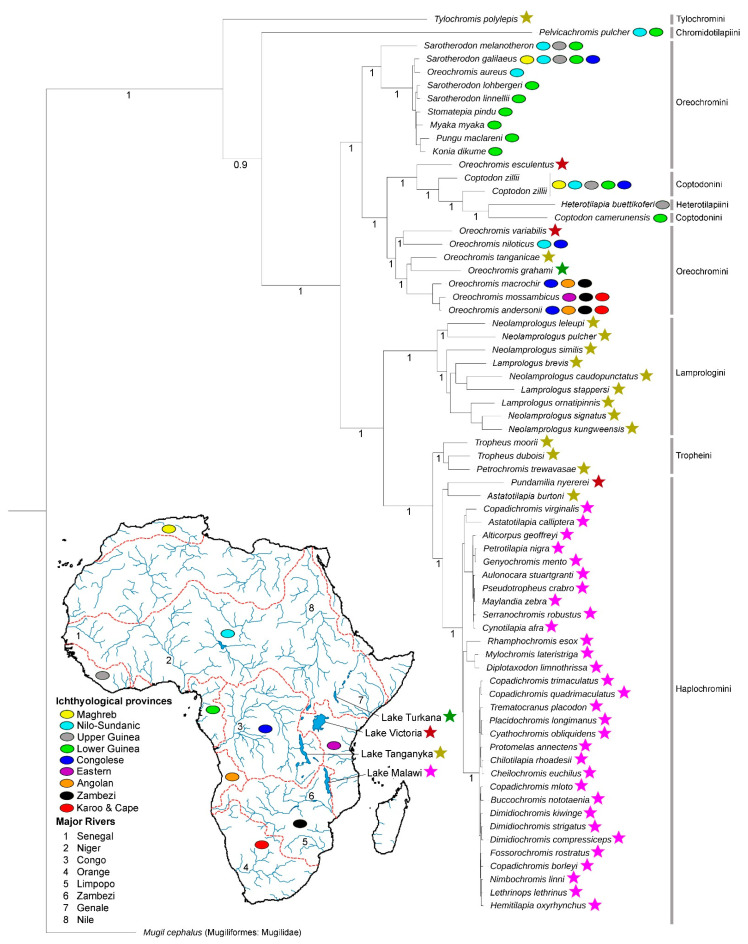
The evolutionary relationship of *C. camerunensis* and other Old-world African Cichlids is depicted by a Bayesian matrilineal tree based on the concatenated sequences of 13 PCGs. The posterior probabilities were superimposed on each node. Different colored stars and oval shapes indicate the unique distribution pattern and adaptability of Cichlids species in Africa.

The mitogenomes-based phylogeny readily delineated all the studied African cichlids using 13 concamerated PCGs. Most of the species showed monophyletic clustering within their respective tribes except for Oreochromini and Coptodinini members. The targeted species, *C. camerunensis* depicted close clustering with *H. buttikoferi* (tribe Heterotilapiini) and showed paraphyletic cladding with its congener, *C. zillii* (Figure 5). The Oreochromini species also showed paraphyletic clustering, and the genus *Oreochromis* revealed a close association with Coptodinini and Heterotilapiini species in the present topology. 

Based on the fossil records, it was evident that cichlid fish first appeared in Mahenge, Tanzania, during the Eocene epoch, about 45 million years ago [100]. Notably, the political boundary of Tanzania shared all three lakes, Lake Victoria, Lake Tanganyka, and Lake Malawi. The present dataset shows *Tylochromis polylepis* (tribe Tylochromini) to be endemic to Lake Tanganyka, placed in the basal node of the current topology (Figure 5). Thus, based on the present topology and the known distribution of Tylochromini species, this study corroborates the early hypothesis of cichlid fishes’ evolution in East Africa [101]. The present topology also reflects the distinct cladding patterns and possible diversification of cichlid fish in different major river basins with respect to the ichthyological provinces and different lakes of Africa.

However, few cichlid species endemic to African lakes (*Oreochromis esculentus* and *Oreochromis variabilis* endemic to Lake Victoria, *Oreochromis tanganicae* endemic to Lake Tanganyka, and *Oreochromis graham* endemic to Lake Turkana) showed a close association with the species distributed in different river basins in the present BA topology (Figure 5). Based on the current BA phylogeny, the present results depicted that the recently evolved cichlid fish classified under the tribes Lamprologini, Tropheini, and Haplochromini are cohesively clustered within their respective lineages with monophyletic clades, as depicted in earlier studies [102,103,104,105]. The present mitogenomic phylogeny showed that the cichlid species under the Haplochromini tribe members are endemic to a rift lake, and Malawi formed a distinct clade. As evidenced previously, the topology also supported the earlier hypothesis and established the youngest species-rich cichlid radiations in the rift lake [106,107,108,109,110]. Owing to the present matrilineal relationship, the present study suggested that more genomic data of cichlid fish from all extant taxonomic lineages and TimeTree calibration with fossils records are required to elucidate the in-depth evolutionary relationship of all old-world cichlids in the African continent.

### 3.6. Geological Opportunities of Old-World Cichlids Radiations

**Figure 6:**
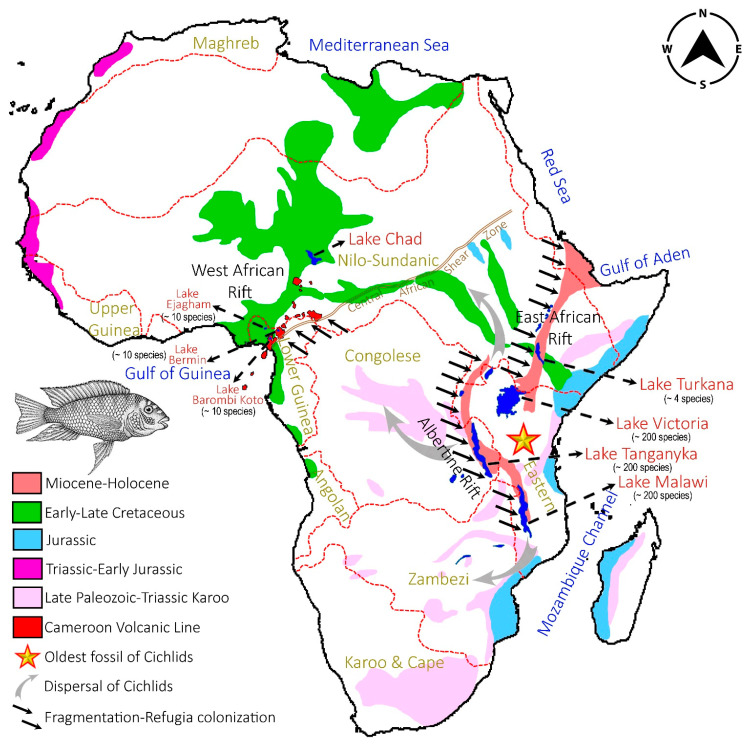
The hypothesized fragmentation–refugia processes generated by African rift systems are depicted on a map along with the potential for the dispersal and colonization of old-world cichlids in various ichthyological provinces and lakes in Africa. The rift systems in eastern, central and western Africa as well as Volcanic Line of Cameroon are illustrated from previous studies [124,127]. Map not to scale and the illustration of cichlid fish were obtained from the web.

Cichlids are one of the largest vertebrate families with one of the highest speciation rates in the world [111]. Several studies have been undertaken in an effort to learn more about their evolution and diversity across continents [53,112]. Although it was previously thought that cichlid fish first appeared in Eastern Africa during the Eocene epoch (~45 Ma) [101,113], a recent genomic timeline revealed that the continental lineages evolved via Gondwanan vicariance (~150 Ma), oceanic dispersal (~70 Ma), and independent colonization (>45 Ma) [53,114]. Furthermore, the diversity and accelerated adaptive radiation of cichlids across the African continent have been reinforced with numerous hypotheses due to their complicated demographic history [25,26,34,75,115,116,117]. Several biological and ecological processes that might influence the tendency of cichlid speciation in different ichthyological provinces and Rift Lakes in Africa have long been addressed [118,119,120]. From ancient times, three main lakes emerged in the East African rift system: Lake Tanganyika (~9–12 Ma), Lake Malawi (~1–2 Ma), and Lake Victoria (≥400 ka) [121]. The Lake Tanganyika is located inside East African Rift’s western branch (Albertine Rift) with two inputs (Ruzizi River from Lake Kivu and Malagarasi River from Tanzania) and one outflow (Lukuga River discharges into the Congo River basin) of riverine systems. Lake Malawi was formed by the opening of the East African rift, which had one inflow (the Ruhuhu River, which rises in Tanzania) and one outflow (the Shire River, which flows into the Zambezi River basin). Lake Victoria, on the other hand, was produced by the uplift of the rift walls of both the East African rift and the Albertine Rift, with one input (Kagera River coming from Lake Rweru) and one outflow (Victoria Nile River empties into Lake Albert) of riverine systems. These ancient Lake systems are home to a primarily rich freshwater species as well as accommodating the footprints of pre-historic early hominin evolution [122,123,124]. The number of cichlid species in Lake Tanganyika is estimated to be 240 [75], with 860 in Lake Malawi [42] and over 500 in Lake Victoria [111]. It can be assumed that the early dispersal of cichlids occurred across different African ichthyological provinces before the active continental rift zone formed 20–25 million years ago and that it was later confined in East African rift lakes due to the formation of various rift systems in Eastern, central, and western Africa linked with geological time (Figure 6). Such ample species diversity, with over 1200 cichlid species, have evolved over the last several million years, and their adaptation in East African rift lakes could be triggered by rift system fragmentation refugia mechanisms [3,125,126].

Aside from East Africa, Western Africa has also accommodated various distinct ichthyofauna, including cichlid species. Cameroon, in particular, is home to multiple endemic fish species in different riverine systems and volcanic lakes, each with its restricted radiation that has been separated by a unique genetic drift. The Cameroon volcanic line, which includes the Gulf of Guinea island and mountain chains on the African mainland, as well as the larger Precambrian Central African Shear Zone, which extends from the Gulf of Guinea to Sudan, generate an unequaled riverine flow in central and western Africa [128]. Furthermore, the Gulf of Guinea mantle plume might have played a key role in the triple junction that formed the Mesozoic African rift system [127,129]. These rift systems have generated several tiny volcanic lakes (Lake Bermin and Lake Barombi Koto) in Cameroon’s southwest area, which are home to several unique cichlid species (Figure 6). The demography of Cameroon was made-up during the break-up of the Gondwana split and was later rejuvenated during the opening of the South Atlantic Ocean during the Jurassic–Cretaceous period and was subsequently shaped by different river basins. The Mbu River, a tributary of the Cross River that runs in southern Nigeria and joins the Mungo River before emptying into the Gulf of Guinea, makes up Lake Bermin’s inflow and outflow. Both Lake Bermin and Lake Barombi Koto are home to many endemic cichlid species (~10 species), which may have been colonized due to the East African rift system [130]. However, Lake Ejagham, located in Cameroon’s southwest area, was formed by groundwater during the last glacial era and is home to six indigenous cichlid species.

The west-central nation of Cameroon significantly contributes to the continent of Africa’s distinctive faunal makeup. Due to Cameroon’s location at the confluence of the Congo Basin and the West African rainforest, several aquatic habitats have converged there. Due to the range of habitats and ecological niches created by such geographic diversity, numerous organisms, including fish populations, have evolved and adapted [131,132]. A variety of habitats, including swiftly running rivers, slowly moving streams, flooded forests, and shallow marshes, are offered by the river systems that traverse Cameroon. Each habitat provides unique conditions that have an impact on how fish species adapt and diversify. Therefore, fish species have developed in this intricate and species-rich habitat, leading to distinct ecological adaptations. Cameroon’s distinct geologic history has resulted in the existence of several indigenous freshwater fish species. The complicated geological processes that molded the area millions of years ago are what have given rise to Cameroon’s unique geological structure. A portion of the broader East African Rift System, the West and Central African rift system is where Cameroon is located. Volcanic eruptions, tectonic movements, shifting landmasses, sedimentation, and erosion over millions of years have formed the varied topography of this nation, leading to its unmatched biogeography. These geological changes have helped to create unique habitats and geographical boundaries, which have aided in the diversification and endemism of species. Over a long period of time, these endemic species have developed and adapted to a particular confined environment or geographic location, giving rise to distinctive traits and genetic diversity. The known geographical distribution of *Coptodon* congeners is distinct from that of other cichlids, with the majority of them being limited to Western Africa. Cameroon is home to 17 of the 32 existing *Coptodon* species. These endemic species are found in Lake Bermin (9 species), Lake Ejagham (4 species), Sanaga River, Wouri River, Nyong River, and Lake Barombi Koto. Based on the observed endemicity, it is reasonable to believe that the West African rift system has had an important influence on the development of *Coptodon* species in Western Africa. From the known distribution of the studied species, *C. camerunensis* in the Wouri River originates at the meeting of the Nkam and Makombe River systems northeast of Yabassi City and empties into the Gulf of Guinea. A current expedition in the Nyong River, however, explains the *C. camerunensis’* expanded range distribution in south-eastern Cameroon. The current study proposes that correct geographical information, prehistoric drainage evolution, coupled with genomic-based cladistics pattern, would clarify a concrete scenario of old-world cichlids development, including *Coptodon* lineages in Africa. This is because of the current distribution and diversification.

## 4. Conclusions

The present study decodes the complete mitogenome of *Coptodon camerunensis*, which are endemic to Cameroon. The structure and variation in different genes could help us to gain knowledge on the mitogenomic evolution of studied species compared with its congener, *Coptodon zillii*. The functions of the mitochondrial genome and its related genes can be inferred from such empirical molecular data. The mitogenome-based Bayesian phylogeny readily delineated all of the investigated African cichlids, including *Coptodon camerunensis,* and revealed paraphyletic clustering with *Coptodon zillii*, in contrast to the earlier hypothesis. However, in order to identify the evolutionary path of *Coptodon* genus members and confirm their systematic classification, additional mitogenomes from West Africa must be generated. Nuclear genome or whole genome data may help with future research on the phylogeny and population genetics of cichlid fishes. The current study also indicates that geological opportunity has a considerable impact on the African continent’s hydroclimate, which has resulted in the diversification and/or colonization of cichlid species in various ichthyological provinces and rift lakes.

## Figures and Tables

**Table 1:** List of annotated mitochondrial genes of *C. camerunensis*.

Genes	Start	End	Strand	Size (bp)	Intergenic Nucleotide	Anti-Codon	Start Codon	Stop Codon
tRNA-Phe (F)	1	69	H	69	0	AAG		
12S rRNA	70	1014	H	945	0			
tRNA-Val (V)	1015	1086	H	72	0	CAT		
16S rRNA	1087	2777	H	1691	0			
tRNA-Leu (L2)	2778	2851	H	74	0	AAT		
ND1	2852	3826	H	975	3		ATG	TAA
tRNA-Ile (I)	3830	3899	H	70	−1	TAG		
tRNA-Gln (Q)	3899	3969	L	71	−1	GTT		
tRNA-Met (M)	3969	4037	H	69	0	TAC		
ND2	4038	5083	H	1046	0		ATG	T--
tRNA-Trp (W)	5084	5155	H	72	1	ACT		
tRNA-Ala (A)	5157	5225	L	69	1	CGT		
tRNA-Asn (N)	5227	5299	L	73	35	TTG		
tRNA-Cys (C)	5335	5400	L	66	0	ACG		
tRNA-Tyr (Y)	5401	5470	L	70	1	ATG		
COI	5472	7067	H	1596	0		GTG	TAA
tRNA-Ser (S2)	7068	7138	L	71	3	AGT		
tRNA-Asp (D)	7142	7214	H	73	5	CTG		
COII	7220	7910	H	691	0		ATG	T--
tRNA-Lys (K)	7911	7984	H	74	1	TTT		
ATP8	7986	8153	H	168	−10		ATG	TAA
ATP6	8144	8826	H	683	0		ATG	TA-
COIII	8827	9610	H	784	0		ATG	T--
tRNA-Gly (G)	9611	9682	H	72	0	CCT		
ND3	9683	10,031	H	349	0		ATG	T--
tRNA-Arg (R)	10,032	10,100	H	69	0	GCT		
ND4L	10,101	10,397	H	297	−7		ATG	TAA
ND4	10,391	11,771	H	1381	0		ATG	T--
tRNA-His (H)	11,772	11,840	H	69	0	GTG		
tRNA-Ser (S1)	11,841	11,907	H	67	4	TCG		
tRNA-Leu (L1)	11,912	11,984	H	73	0	GAT		
ND5	11,985	13,823	H	1839	−4		ATA	TAA
ND6	13,820	14,341	L	522	0		ATG	TAA
tRNA-Glu (E)	14,342	14,410	L	69	4	CTT		
Cyt b	14,415	15,555	H	1141	0		ATG	T--
tRNA-Thr (T)	15,556	15,627	H	72	0	TGT		
tRNA-Pro (P)	15,628	15,697	L	70	−69	GGT		
Control region	15,629	16,557		929				

**Table 2:** Comparative nucleotide composition: AT and GC Skews of two Coptodonini species mitogenomes.

Species Name	Size (bp)	A%	T%	G%	C%	A + T%	AT-Skew	GC-Skew
***Complete mitogenome***								
*C. camerunensis* (OQ696044)	16,557	27.124	25.506	16.380	30.990	52.630	0.031	−0.308
*C. zillii* (MW194077)	16,551	27.09	25.67	16.57	30.67	52.760	0.027	−0.299
*C. zillii* (KM658974)	16,619	27.26	25.78	16.29	30.68	53.040	0.028	−0.306
***PCGs***								
*C. camerunensis* (OQ696044)	11,472	24.076	27.510	16.169	32.243	51.586	−0.067	−0.332
*C. zillii* (MW194077)	11,472	24.16	27.45	16.29	32.1	51.61	−0.064	−0.327
*C. zillii* (KM658974)	11,472	24.39	27.59	15.91	32.11	51.98	−0.062	−0.337
***rRNAs***								
*C. camerunensis* (OQ696044)	2636	32.132	20.902	20.599	26.365	53.034	0.212	−0.123
*C. zillii* (MW194077)	2638	32.18	20.96	20.51	26.35	53.14	0.211	−0.125
*C. zillii* (KM658974)	2637	32.01	20.89	20.59	26.51	52.9	0.210	−0.126
***tRNAs***								
*C. camerunensis* (OQ696044)	1554	28.185	26.512	23.487	21.814	54.697	0.031	0.037
*C. zillii* (MW194077)	1554	28.19	26.64	23.42	21.75	54.83	0.028	0.037
*C. zillii* (KM658974)	1553	28.01	26.92	23.63	21.44	54.93	0.020	0.049
***CRs***								
*C. camerunensis* (OQ696044)	929	32.292	30.570	14.639	22.497	62.863	0.027	−0.212
*C. zillii* (MW194077)	852	32.16	32.28	15.26	20.31	64.44	−0.002	−0.142
*C. zillii* (KM658974)	924	31.6	32.9	14.5	21	64.5	−0.020	−0.183

## Data Availability

The genome sequence data that support the findings of this study are openly available in GenBank of NCBI at https://www.ncbi.nlm.nih.gov (accessed on 15 July 2023), under the accession no. OQ696044.

## Supplementary Materials

The following supporting information can be downloaded at: https://www.mdpi.com/article/10.3390/genes14081591/s1, Table S1. Dataset of African cichlids for the present phylogenetic study (Accessed on 19 April 2023); Table S2. Comparison of the intergenic nucleotides of two *Coptodon* species mitogenomes; Table S3. Start and stop codons of all 13 PCGS in two *Coptodon* species mitogenomes.

## References

[1] Fricke R., Eschmeyer W.N., Van der Laan R. (2022). Eschmeyer’s Catalog of Fishes: Genera, Species.

[2] Baker M.R., Ballantyne R. (2015). The role of aquarium fish in conservation education. Conserv. Biol..

[3] Santos M.E., Lopes J.F., Kratochwil C.F. (2023). East African cichlid fishes. EvoDevo.

[4] Sefc K.M. (2011). Mating and Parental Care in Lake Tanganyika’s Cichlids. Int. J. Evol. Biol..

[5] Tsuboi M., Gonzalez-Voyer A., Kolm N. (2014). Phenotypic integration of brain size and head morphology in Lake Tanganyika Cichlids. BMC Evol. Biol..

[6] Navon D., Olearczyk N., Albertson R.C. (2017). Genetic and developmental basis for fin shape variation in African cichlid fishes. Mol. Ecol..

[7] York R.A., Patil C., Abdilleh K., Johnson Z.V., Conte M.A., Genner M.J., McGrath P.T., Fraser H.B., Fernald R.D., Streelman J.T. (2018). Behavior-dependent cis regulation reveals genes and pathways associated with bower building in cichlid fishes. Proc. Natl. Acad. Sci. USA.

[8] Edgley D.E., Genner M.J. (2019). Adaptive Diversification of the Lateral Line System during Cichlid Fish Radiation. iScience.

[9] Urban S., Nater A., Meyer A., Kratochwil C.F. (2021). Different Sources of Allelic Variation Drove Repeated Color Pattern Divergence in Cichlid Fishes. Mol. Biol. Evol..

[10] Feller A.F., Haesler M.P., Peichel C.L., Seehausen O. (2020). Genetic architecture of a key reproductive isolation trait differs between sympatric and nonsympatric sister species of Lake Victoria cichlids. Proc. R Soc. B Biol. Sci..

[11] Salzburger W., Baric S., Sturmbauer C. (2002). Speciation via introgressive hybridization in East African cichlids?. Mol. Ecol..

[12] Genner M.J., Turner G.F. (2012). Ancient hybridization and phenotypic novelty within Lake Malawi’s cichlid fish radiation. Mol. Biol. Evol..

[13] Meier J.I., Marques D.A., Mwaiko S., Wagner C.E., Excoffier L., Seehausen O. (2017). Ancient hybridization fuels rapid cichlid fish adaptive radiations. Nat. Commun..

[14] Svardal H., Quah F.X., Malinsky M., Ngatunga B.P., Miska E.A., Salzburger W., Genner M.J., Turner G.F., Durbin R. (2020). Ancestral Hybridization Facilitated Species Diversification in the Lake Malawi Cichlid Fish Adaptive Radiation. Mol. Biol. Evol..

[15] Chakrabarty P. (2006). Systematics and historical biogeography of Greater Antillean Cichlidae. Mol. Phylogenet. Evol..

[16] Leo Smith W., Chakrabarty P., Sparks J. (2008). Phylogeny, taxonomy, and evolution of Neotropical cichlids (Teleostei: Cichlidae: Cichlinae). Cladistics.

[17] López-Fernández H., Winemiller K.O., Honeycutt R.L. (2010). Multilocus phylogeny and rapid radiations in Neotropical cichlid fishes (Perciformes: Cichlidae: Cichlinae). Mol. Phylogenet. Evol..

[18] Ilves K.L., Torti D., López-Fernández H. (2018). Exon-based phylogenomics strengthens the phylogeny of Neotropical cichlids and identifies remaining conflicting clades (Cichliformes: Cichlidae: Cichlinae). Mol. Phylogenet. Evol..

[19] Fan S., Elmer K.R., Meyer A. (2012). Genomics of adaptation and speciation in cichlid fishes: Recent advances and analyses in African and Neotropical lineages. Philos. Trans. R Soc. Lond. B Biol. Sci..

[20] Arbour J.H., López-Fernández H. (2016). Continental cichlid radiations: Functional diversity reveals the role of changing ecological opportunity in the Neotropics. Proc. Biol. Sci..

[21] Burress E.D., Piálek L., Casciotta J., Almirón A., Říčan O. (2023). Rapid Parallel Morphological and Mechanical Diversification of South American Pike Cichlids (*Crenicichla*). Syst. Biol..

[22] Verheyen E., Salzburger W., Snoeks J., Meyer A. (2003). Origin of the superflock of cichlid fishes from Lake Victoria, East Africa. Science.

[23] Sparks J.S., Smith W.L. (2004). Phylogeny and biogeography of cichlid fishes (Teleostei: Perciformes: Cichlidae). Cladistics.

[24] Santos M.E., Salzburger W. (2012). Evolution. How cichlids diversify. Science.

[25] Brawand D., Wagner C.E., Li Y.I., Malinsky M., Keller I., Fan S., Simakov O., Ng A.Y., Lim Z.W., Bezault E. (2014). The genomic substrate for adaptive radiation in African cichlid fish. Nature.

[26] Malinsky M., Salzburger W. (2016). Environmental context for understanding the iconic adaptive radiation of cichlid fishes in Lake Malawi. Proc. Natl. Acad. Sci. USA.

[27] Sparks J.S. (2004). Molecular phylogeny and biogeography of the Malagasy and South Asian cichlids (Teleostei: Perciformes: Cichlidae). Mol. Phylogenet. Evol..

[28] Trape S. (2016). A new cichlid fish in the Sahara: The Ounianga Serir lakes (Chad), a biodiversity hotspot in the desert. C. R. Biol..

[29] Altner M., Ruthensteiner B., Reichenbacher B. (2020). New haplochromine cichlid from the upper Miocene (9–10 MYA) of Central Kenya. BMC Evol. Biol..

[30] Allender C.J., Seehausen O., Knight M.E., Turner G.F., Maclean N. (2003). Divergent selection during speciation of Lake Malawi cichlid fishes inferred from parallel radiations in nuptial coloration. Proc. Natl. Acad. Sci. USA.

[31] Conith M.R., Hu Y., Conith A.J., Maginnis M.A., Webb J.F., Albertson R.C. (2018). Genetic and developmental origins of a unique foraging adaptation in a Lake Malawi cichlid genus. Proc. Natl. Acad. Sci. USA.

[32] Joyce D.A., Lunt D.H., Bills R., Turner G.F., Katongo C., Duftner N., Sturmbauer C., Seehausen O. (2005). An extant cichlid fish radiation emerged in an extinct Pleistocene Lake. Nature.

[33] Lyons R.P., Scholz C.A., Cohen A.S., King J.W., Brown E.T., Ivory S.J., Johnson T.C., Deino A.L., Reinthal P.N., McGlue M.M. (2015). Continuous 1.3-million-year record of East African hydroclimate, and implications for patterns of evolution and biodiversity. Proc. Natl. Acad. Sci. USA.

[34] Ivory S.J., Blome M.W., King J.W., McGlue M.M., Cole J.E., Cohen A.S. (2016). Environmental change explains cichlid adaptive radiation at Lake Malawi over the past 1.2 million years. Proc. Natl. Acad. Sci. USA.

[35] Weber A.A., Rajkov J., Smailus K., Egger B., Salzburger W. (2021). Speciation dynamics and extent of parallel evolution along a lake-stream environmental contrast in African cichlid fishes. Sci. Adv..

[36] Won Y.J., Sivasundar A., Wang Y., Hey J. (2005). On the origin of Lake Malawi cichlid species: A population genetic analysis of divergence. Proc. Natl. Acad. Sci. USA.

[37] Bezault E., Mwaiko S., Seehausen O. (2011). Population genomic tests of models of adaptive radiation in Lake Victoria region cichlid fish. Evolution.

[38] Keller I., Wagner C.E., Greuter L., Mwaiko S., Selz O.M., Sivasundar A., Wittwer S., Seehausen O. (2013). Population genomic signatures of divergent adaptation, gene flow and hybrid speciation in the rapid radiation of Lake Victoria cichlid fishes. Mol. Ecol..

[39] Alter S.E., Munshi-South J., Stiassny M.L. (2017). Genomewide SNP data reveal cryptic phylogeographic structure and microallopatric divergence in a rapids-adapted clade of cichlids from the Congo River. Mol. Ecol..

[40] Hulsey C.D., Zheng J., Faircloth B.C., Meyer A., Alfaro M.E. (2017). Phylogenomic analysis of Lake Malawi cichlid fishes: Further evidence that the three-stage model of diversification does not fit. Mol. Phylogenet. Evol..

[41] Irisarri I., Singh P., Koblmüller S., Torres-Dowdall J., Henning F., Franchini P., Fischer C., Lemmon A.R., Lemmon E.M., Thallinger G.G. (2018). Phylogenomics uncovers early hybridization and adaptive loci shaping the radiation of Lake Tanganyika cichlid fishes. Nat. Commun..

[42] Malinsky M., Svardal H., Tyers A.M., Miska E.A., Genner M.J., Turner G.F., Durbin R. (2018). Whole-genome sequences of Malawi cichlids reveal multiple radiations interconnected by gene flow. Nat. Ecol. Evol..

[43] Astudillo-Clavijo V., Stiassny M.L.J., Ilves K.L., Musilova Z., Salzburger W., López-Fernández H. (2023). Exon-based Phylogenomics and the Relationships of African Cichlid Fishes: Tackling the Challenges of Reconstructing Phylogenies with Repeated Rapid Radiations. Syst. Biol..

[44] Albertson R.C., Markert J.A., Danley P.D., Kocher T.D. (1999). Phylogeny of a rapidly evolving clade: The cichlid fishes of Lake Malawi, East Africa. Proc. Natl. Acad. Sci. USA.

[45] Seehausen O., Koetsier E., Schneider M.V., Chapman L.J., Chapman C.A., Knight M.E., Turner G.F., van Alphen J.J., Bills R. (2003). Nuclear markers reveal unexpected genetic variation and a Congolese-Nilotic origin of the Lake Victoria cichlid species flock. Proc. Biol. Sci..

[46] Kassam D., Seki S., Horic M., Yamaoka K. (2006). Nuclear markers reveal that inter-lake cichlids’ similar morphologies do not reflect similar genealogy. Mol. Phylogenet. Evol..

[47] Won Y.J., Wang Y., Sivasundar A., Raincrow J., Hey J. (2006). Nuclear gene variation and molecular dating of the cichlid species flock of Lake Malawi. Mol. Biol. Evol..

[48] Koblmüller S., Egger B., Sturmbauer C., Sefc K.M. (2010). Rapid radiation, ancient incomplete lineage sorting and ancient hybridization in the endemic Lake Tanganyika cichlid tribe Tropheini. Mol. Phylogenet. Evol..

[49] Meyer B.S., Matschiner M., Salzburger W. (2015). A tribal level phylogeny of Lake Tanganyika cichlid fishes based on a genomic multi-marker approach. Mol. Phylogenet. Evol..

[50] Takahashi T., Sota T. (2016). A robust phylogeny among major lineages of the East African cichlids. Mol. Phylogenet. Evol..

[51] Meyer B.S., Matschiner M., Salzburger W. (2017). Disentangling Incomplete Lineage Sorting and Introgression to Refine Species-Tree Estimates for Lake Tanganyika Cichlid Fishes. Syst. Biol..

[52] Schedel F.D.B., Musilova Z., Schliewen U.K. (2019). East African cichlid lineages (Teleostei: Cichlidae) might be older than their ancient host lakes: New divergence estimates for the east African cichlid radiation. BMC Evol. Biol..

[53] Matschiner M., Böhne A., Ronco F., Salzburger W. (2020). The genomic timeline of cichlid fish diversification across continents. Nat. Commun..

[54] He A., Luo Y., Yang H., Liu L., Li S., Wang C. (2011). Complete mitochondrial DNA sequences of the Nile tilapia (*Oreochromis niloticus*) and Blue tilapia (*Oreochromis aureus*): Genome characterization and phylogeny applications. Mol. Biol. Rep..

[55] Bbole I., Zhao J.L., Tang S.J., Katongo C. (2018). Mitochondrial genome annotation and phylogenetic placement of *Oreochromis andersonii* and *O. macrochir* among the cichlids of southern Africa. PLoS ONE..

[56] Boore J.L. (1999). Animal mitochondrial genomes. Nucleic Acids Res..

[57] Galtier N., Nabholz B., Glemin S., Hurst G.D. (2009). Mitochondrial DNA as a marker of molecular diversity: A reappraisal. Mol. Ecol..

[58] Miya M., Nishida M. (1999). Organization and variation of the mitochondrial genome of fishes. J. Mol. Evol..

[59] Miya M., Kawaguchi A., Nishida M. (2001). Mitogenomic exploration of higher teleostean phylogenies: A case study for moderate-scale evolutionary genomics with 38 newly determined complete mitochondrial DNA sequences. Mol. Biol. Evol..

[60] Miya M., Takeshima H., Endo H., Ishiguro N.B., Inoue J.G., Mukai T., Satoh T.P., Yamaguchi M., Kawaguchi A., Mabuchi K. (2003). Major patterns of higher teleostean phylogenies: A new perspective based on 100 complete mitochondrial DNA sequences. Mol. Phylogenet. Evol..

[61] Roberts T.R. (1982). Systematic revision of the old-world freshwater fish family Cichlidae. Bull. Mus. Comp. Zool..

[62] Meyer A., Kocher T.D., Basasibwaki P., Wilson A.C. (1990). Monophyletic origin of Lake Victoria cichlid fishes suggested by mitochondrial DNA sequences. Nature.

[63] Dunz A.R., Schliewen U.K. (2013). Molecular phylogeny and revised classification of the haplotilapiine cichlid fishes formerly referred to as “Tilapia”. Mol. Phylogenet. Evol..

[64] Konings A. (2015). Cichlids from West Africa: A review and comments on their nomenclature, taxonomy, and distribution. J. Fish Biol..

[65] Falade M.O., Opene A.J., Benson O. (2016). DNA barcoding of *Clarias gariepinus*, *Coptodon zillii* and *Sarotherodon melanotheron* from Southwestern Nigeria. F1000Research.

[66] Soliman T., Aly W., Fahim R.M., Berumen M.L., Jenke-Kodama H., Bernardi G. (2017). Comparative population genetic structure of redbelly tilapia (*Coptodon zillii* (Gervais, 1848)) from three different aquatic habitats in Egypt. Ecol. Evol..

[67] Agnèse J.F., Louizi H., Gilles A., Berrada Rkhami O., Benhoussa A., Qninba A., Pariselle A. (2018). A euryhaline fish, lost in the desert: The unexpected metapopulation structure of *Coptodon guineensis* (Günther, 1862) in the Sebkha of Imlili. C. R. Biol..

[68] Taylor M.I., van Riemsdijk I. (2020). Hidden diversity in West African Coptodon: Description of three new species (Teleostei, Cichlidae). ZooKeys.

[69] Kinaro Z.O., Xue L., Volatiana J.A. (2016). Complete mitochondrial DNA sequences of the Victoria tilapia (*Oreochromis variabilis*) and Redbelly Tilapia (*Tilapia zilli*): Genome characterization and phylogeny analysis. Mitochondrial DNA. Part A DNA Mapp. Seq. Anal..

[70] Fiteha Y.G., Rashed M.A., Ali R.A.M., Magdy M. (2023). Characterization and phylogenetic analysis of the complete mitochondrial genome of Mango tilapia (*Sarotherodon galilaeus*: Cichlidae). Mol Biol Rep..

[71] IUCN (2023). The IUCN Red List of Threatened Species. Version 2022-2..

[72] Moritz C. (1994). Defining “evolutionarily significant units” for conservation. Trends Ecol. Evol..

[73] Matschiner M., Musilová Z., Barth J.M., Starostová Z., Salzburger W., Steel M., Bouckaert R. (2017). Bayesian Phylogenetic Estimation of Clade Ages Supports Trans-Atlantic Dispersal of Cichlid Fishes. Syst. Biol..

[74] Matschiner M. (2019). Gondwanan vicariance or trans-Atlantic dispersal of cichlid fishes: A review of the molecular evidence. Hydrobiologia.

[75] Ronco F., Matschiner M., Böhne A., Boila A., Büscher H.H., El Taher A., Indermaur A., Malinsky M., Ricci V., Kahmen A. (2021). Drivers and dynamics of a massive adaptive radiation in cichlid fishes. Nature.

[76] Lamboj A. (2004). The Cichlid Fishes of Western Africa.

[77] Baldwin C.C., Mounts J.H., Smith D.G., Weight L.A. (2009). Genetic identification and color descriptions of early life-history stages of Belizean *Phaeoptyx* and *Astrapogon* (Teleostei: Apogonidae) with comments on identification of adult *Phaeoptyx*. Zootaxa.

[78] Kide N.G., Dunz A., Agnèse J.F., Dilyte J., Pariselle A., Carneiro C., Correia E., Brito J.C., Yarba L.O., Kone Y. (2016). Cichlids of the Banc d’Arguin National Park, Mauritania: Insight into the diversity of the genus *Coptodon*. J. Fish Biol..

[79] Bernt M., Donath A., Jühling F., Externbrink F., Florentz C., Fritzsch G., Pütz J., Middendorf M., Stadler P.F. (2013). MITOS: Improved de novo Metazoan Mitochondrial Genome Annotation. Mol. Phylogenet. Evol..

[80] Iwasaki W., Fukunaga T., Isagozawa R., Yamada K., Maeda Y., Satoh T.P., Sado T., Mabuchi K., Takeshima H., Miya M. (2013). MitoFish and MitoAnnotator: A mitochondrial genome database of fish with an accurate and automatic annotation pipeline. Mol. Biol. Evol..

[81] Kumar S., Stecher G., Li M., Knyaz C., Tamura K. (2018). MEGA X: Molecular Evolutionary Genetics Analysis across computing platforms. Mol. Biol. Evol..

[82] Perna N.T., Kocher T.D. (1995). Patterns of nucleotide composition at fourfold degenerate sites of animal mitochondrial genomes. J. Mol. Evol..

[83] Laslett D., Canbäck B. (2008). ARWEN, a program to detect tRNA genes in metazoan mitochondrial nucleotide sequences. Bioinformatics.

[84] Lowe T.M., Chan P.P. (2016). tRNAscan-SE On-line: Integrating search and context for analysis of transfer RNA genes. Nucleic Acids Res..

[85] Thompson J.D., Gibson T.J., Plewniak F., Jeanmougin F., Higgins D.G. (1997). The CLUSTAL_X windows interface: Flexible strategies for multiple sequence alignment aided by quality analysis tools. Nucleic Acids Res..

[86] Benson G. (1999). Tandem repeats finder: A program to analyze DNA sequences. Nucleic Acids Res..

[87] Vences M., Miralles A., Brouillet S., Ducasse J., Fedosov A., Kharchev V., Kostadinov I., Kumari S., Patmanidis S., Scherz M.D. (2021). iTaxoTools 0.1: Kickstarting a specimen-based software toolkit for taxonomists. Megataxa.

[88] Ronquist F., Huelsenbeck J.P. (2003). MrBayes 3: Bayesian phylogenetic inference under mixed models. Bioinformatics.

[89] Letunic I., Bork P. (2007). Interactive Tree of Life (iTOL): An online tool for phylogenetic tree display and annotation. Bioinformatics.

[90] Kundu S., Binarao J.D., De Alwis P.S., Kim A.R., Lee S.R., Andriyono S., Gietbong F.Z., Kim H.W. (2023). First Mitogenome of Endangered *Enteromius thysi* (Actinopterygii: Cypriniformes: Cyprinidae) from Africa: Characterization and Phylogeny. Fishes.

[91] De Alwis P.S., Kundu S., Gietbong F.Z., Amin M.H.F., Lee S.R., Kim H.W., Kim A.R. (2023). Mitochondriomics of *Clarias* Fishes (Siluriformes: Clariidae) with a New Assembly of *Clarias camerunensis*: Insights into the Genetic Characterization and Diversification. Life.

[92] Wang X., Wang Y., Zhang Y., Yu H., Tong J. (2016). Evolutionary analysis of cyprinid mitochondrial genomes: Remarkable variation and strong adaptive evolution. Front. Genet..

[93] Ojala D., Montoya J., Attardi G. (1981). tRNA punctuation model of RNA processing in human mitochondria. Nature.

[94] Foote A.D., Morin P.A., Durban J.W., Pitman R.L., Wade P., Willerslev E., Gilbert M.T., da Fonseca R.R. (2011). Positive selection on the killer whale mitogenome. Biol. Lett..

[95] Hill J., Enbody E.D., Pettersson M.E., Sprehn C.G., Bekkevold D., Folkvord A., Laikre L., Kleinau G., Scheerer P., Andersson L. (2019). Recurrent convergent evolution at amino acid residue 261 in fish rhodopsin. Proc. Natl. Acad. Sci. USA.

[96] Sato N.S., Hirabayashi N., Agmon I., Yonath A., Suzuki T. (2006). Comprehensive genetic selection revealed essential bases in the peptidyl-transferase center. Proc. Natl. Acad. Sci. USA.

[97] Satoh T.P., Miya M., Mabuchi K., Nishida M. (2016). Structure and variation of the mitochondrial genome of fishes. BMC Genom..

[98] Fiteha Y.G., Magdy M. (2022). The Evolutionary Dynamics of the Mitochondrial tRNA in the Cichlid Fish Family. Biology.

[99] Varani G., McClain W.H. (2000). The G-U wobble base pair: A fundamental building block of RNA structure crucial to RNA function in diverse biological systems. EMBO Rep..

[100] Murray A.M. (2000). Eocene cichlid fishes from Tanzania, East Africa. J. Vertebr. Paleontol..

[101] Murray A.M. (2001). The oldest fossil cichlids (Teleostei: Perciformes): Indication of a 45-million-year-old species flock. Proc. Biol. Sci..

[102] Salzburger W., Meyer A., Baric S., Verheyen E., Sturmbauer C. (2002). Phylogeny of the Lake Tanganyika cichlid species flock and its relationship to the Central and East African haplochromine cichlid fish faunas. Syst. Biol..

[103] Koblmüller S., Schliewen U.K., Duftner N., Sefc K.M., Katongo C., Sturmbauer C. (2008). Age and spread of the haplochromine cichlid fishes in Africa. Mol. Phylogenet. Evol..

[104] Sturmbauer C., Salzburger W., Duftner N., Schelly R., Koblmüller S. (2010). Evolutionary history of the Lake Tanganyika cichlid tribe Lamprologini (Teleostei: Perciformes) derived from mitochondrial and nuclear DNA data. Mol. Phylogenet. Evol..

[105] Wanek K.A., Sturmbauer C. (2015). Form, function and phylogeny: Comparative morphometrics of Lake Tanganyika’s cichlid tribe Tropheini. Zool. Scr..

[106] Hermann C.M., Sefc K.M., Koblmüller S. (2011). Ancient origin and recent divergence of a haplochromine cichlid lineage from isolated water bodies in the East African Rift system. J. Fish Biol..

[107] Meyer B.S., Indermaur A., Ehrensperger X., Egger B., Banyankimbona G., Snoeks J., Salzburger W. (2015). Back to Tanganyika: A case of recent trans-species-flock dispersal in East African haplochromine cichlid fishes. R. Soc. Open Sci..

[108] Bloomquist R.F., Fowler T.E., Sylvester J.B., Miro R.J., Streelman J.T. (2017). A compendium of developmental gene expression in Lake Malawi cichlid fishes. BMC Dev. Biol..

[109] Moser F.N., van Rijssel J.C., Mwaiko S., Meier J.I., Ngatunga B., Seehausen O. (2018). The onset of ecological diversification 50 years after colonization of a crater lake by haplochromine cichlid fishes. Proc. Biol. Sci..

[110] Olave M., Meyer A. (2020). Implementing Large Genomic Single Nucleotide Polymorphism Data Sets in Phylogenetic Network Reconstructions: A Case Study of Particularly Rapid Radiations of Cichlid Fish. Syst. Biol..

[111] McGee M.D., Borstein S.R., Meier J.I., Marques D.A., Mwaiko S., Taabu A., Kishe M.A., O’Meara B., Bruggmann R., Excoffier L. (2020). The ecological and genomic basis of explosive adaptive radiation. Nature.

[112] Salzburger W. (2018). Understanding explosive diversification through cichlid fish genomics. Nat. Rev. Genet..

[113] Schwarzer J., Misof B., Tautz D., Schliewen U.K. (2009). The root of the East African cichlid radiations. BMC Evol. Biol..

[114] Friedman M., Keck B.P., Dornburg A., Eytan R.I., Martin C.H., Hulsey C.D., Wainwright P.C., Near T.J. (2013). Molecular and fossil evidence place the origin of cichlid fishes long after Gondwanan rifting. Proc. Biol. Sci..

[115] Fruciano C., Franchini P., Kovacova V., Elmer K.R., Henning F., Meyer A. (2016). Genetic linkage of distinct adaptive traits in sympatrically speciating crater lake cichlid fish. Nat. Commun..

[116] Ronco F., Salzburger W. (2016). Speciation: Genomic Archipelagos in a Crater Lake. Curr. Biol..

[117] Xiong P., Hulsey C.D., Fruciano C., Wong W.Y., Nater A., Kautt A.F., Simakov O., Pippel M., Kuraku S., Meyer A. (2021). The comparative genomic landscape of adaptive radiation in crater lake cichlid fishes. Mol. Ecol..

[118] Day J.J., Cotton J.A., Barraclough T.G. (2008). Tempo and mode of diversification of Lake Tanganyika cichlid fishes. PLoS ONE.

[119] Genner M.J., Ngatunga B.P., Mzighani S., Smith A., Turner G.F. (2015). Geographical ancestry of Lake Malawi’s cichlid fish diversity. Biol. Lett..

[120] Meier J.I., Marques D.A., Wagner C.E., Excoffier L., Seehausen O. (2018). Genomics of Parallel Ecological Speciation in Lake Victoria Cichlids. Mol. Biol. Evol..

[121] Tryon C.A., Faith J.T., Peppe D.J., Beverly E.J., Blegen N., Blumenthal S.A., Chritz K.L., Driese S.G., Patterson D., Sharp W.D. (2016). The Pleistocene prehistory of the Lake Victoria basin. Quat. Int..

[122] Maslin M.A., Brierley C.M., Milner A.M., Shultz S., Trauth M.H., Wilson K.E. (2014). East African climate pulses and early human evolution. Quat. Sci. Rev..

[123] Bibi F., Pante M., Souron A., Stewart K., Varela S., Werdelin L., Boisserie J.R., Fortelius M., Hlusko L., Njau J. (2018). Paleoecology of the Serengeti during the Oldowan-Acheulean transition at Olduvai Gorge, Tanzania: The mammal and fish evidence. J. Hum. Evol..

[124] Joordens J.C.A., Feibel C.S., Vonhof H.B., Schulp A.S., Kroon D. (2019). Relevance of the eastern African coastal forest for early hominin biogeography. J. Hum. Evol..

[125] Ring U., Albrecht C., Schrenk F., Hoorn M.C., Perrigo A., Antonelli A. (2018). The east African rift system tectonics, climate and biodiversity. Mountains, Climate and Biodiversity.

[126] Nakamura H., Aibara M., Kajitani R., Mrosso H.D.J., Mzighani S.I., Toyoda A., Itoh T., Okada N., Nikaido M. (2021). Genomic Signatures for Species-Specific Adaptation in Lake Victoria Cichlids Derived from Large-Scale Standing Genetic Variation. Mol. Biol. Evol..

[127] Marzoli A., Piccirillo E.M., Renne P.R., Bellieni G., Iacumin M., Nyobe J.B., Tongwa A.T. (2000). The Cameroon Volcanic Line Revisited: Petrogenesis of continental basaltic magmas from lithospheric and asthenospheric mantle sources. J. Petrol..

[128] Stankiewicz J., de Wit M.J. (2006). A proposed drainage evolution model for Central Africa-did the Congo flow east?. J. Afr. Earth Sci..

[129] Min G., Hou G. (2019). Mechanism of the Mesozoic African rift system: Paleostress field modeling. J. Geodyn..

[130] Musilova Z., Indermaur A., Bitja-Nyom A.R., Omelchenko D., Kłodawska M., Albergati L., Remišová K., Salzburger W. (2019). Evolution of the visual sensory system in cichlid fishes from crater lake Barombi Mbo in Cameroon. Mol. Ecol..

[131] Burress E.D., Tan M. (2017). Ecological opportunity alters the timing and shape of adaptive radiation. Evolution.

[132] Carruthers M., Edgley D.E., Saxon A.D., Gabagambi N.P., Shechonge A., Miska E.A., Durbin R., Bridle J.R., Turner G.F., Genner M.J. (2022). Ecological Speciation Promoted by Divergent Regulation of Functional Genes Within African Cichlid Fishes. Mol. Biol. Evol..

