# Supplementary material for: Mitogenomic Characterization of Cameroonian Endemic Coptodon camerunensis (Cichliformes: Cichlidae) and Matrilineal Phylogeny of Old-World Cichlids
Source: Genes (Basel). 2023 Aug 6;14(8):1591. doi: 10.3390/genes14081591 (PMC10454717; doi:10.3390/genes14081591)
Supplement: Supplementary file 1 [file genes-14-01591-s001.zip › Table S1.pdf]

Table S1. Dataset of African cichlids for the present phylogenetic study (Accessed on 19 April 2023).

Sl. No.	Species	Tribe	Accession No.	Length (bp)
1	*Coptodon camerunensis*	Coptodonini	OQ696044	16,557
2	*Coptodon zillii*	Coptodonini	MW194077	16,551
3	*Coptodon zillii*	Coptodonini	KM658974	16,619
4	*Lamprologus ornatipinnis*	Lamprologini	OQ076695	16,585
5	*Neolamprologus similis*	Lamprologini	OP930815	16,580
6	*Lamprologus brevis*	Lamprologini	OP930818	16,586
7	*Neolamprologus leleupi*	Lamprologini	OP930817	16,562
8	*Neolamprologus caudopunctatus*	Lamprologini	OP930816	16,586
9	*Copadichromis borleyi*	Haplochromini	OQ558013	16,581
10	*Neolamprologus kungweensis*	Lamprologini	OP805601	16,587
11	*Lamprologus stappersi*	Lamprologini	OP805600	16,582
12	*Oreochromis grahami*	Oreochromini	ON921056	16,626
13	*Sarotherodon galilaeus*	Oreochromini	MW046257	16,630
14	*Stomatepia pindu*	Oreochromini	MK170265	16,636
15	*Sarotherodon lohbergeri*	Oreochromini	MK170264	16,634
16	*Konia dikume*	Oreochromini	MK170263	16,618
17	*Myaka myaka*	Oreochromini	MK170262	16,626
18	*Pungu maclareni*	Oreochromini	MK170261	16,565
19	*Sarotherodon linnellii*	Oreochromini	MK170260	16,634
20	*Oreochromis macrochir*	Oreochromini	MG603675	16,644
21	*Oreochromis andersonii*	Oreochromini	MG603674	16,642
22	*Cyathochromis obliquidens*	Haplochromini	MF033354	16,581
23	*Chilotilapia rhoadesii*	Haplochromini	MF033353	16,580
24	*Copadichromis trimaculatus*	Haplochromini	MF033352	16,581
25	*Lethrinops lethrinus*	Haplochromini	KX595334	16,582
26	*Serranochromis robustus*	Haplochromini	KX595333	16,583
27	*Buccochromis nototaenia*	Haplochromini	KX631426	16,580
28	*Dimidiochromis kiwinge*	Haplochromini	KX266758	16,581
29	*Copadichromis quadrimaculatus*	Haplochromini	KX272653	16,583
30	*Copadichromis mloto*	Haplochromini	KX196155	16,583
31	*Copadichromis virginalis*	Haplochromini	KU144677	16,704
32	*Mylochromis lateristriga*	Haplochromini	KU056478	16,576
33	*Aulonocara stuartgranti*	Haplochromini	KT943516	16,669
34	*Placidochromis longimanus*	Haplochromini	KT309044	16,581
35	*Fossorochromis rostratus*	Haplochromini	KT290557	16,581
36	*Alticorpus geoffreyi*	Haplochromini	KT277287	16,578
37	*Pundamilia nyererei*	Haplochromini	KT222896	16,761
38	*Protomelas annectens*	Haplochromini	KT188786	16,583
39	*Maylandia zebra*	Haplochromini	KT221043	16,582
40	*Astatotilapia burtoni*	Haplochromini	KP641358	16,583
41	*Oreochromis variabilis*	Oreochromini	KM658973	16,626
42	*Oreochromis esculentus*	Oreochromini	KM654981	16,622
43	*Heterotilapia buettikoferi*	Heterotilapiini	KF866133	16,577
44	*Cheilochromis euchilus*	Haplochromini	JN252050	16,582
45	*Tropheus moorii*	Tropheini	HE961975	16,590

46	*Petrochromis trewavasae*	Tropheini	HE961974	16,588
47	*Cynotilapia afra*	Haplochromini	JN628861	16,585
48	*Rhamphochromis esox*	Haplochromini	JN628860	16,583
49	*Genyochromis mento*	Haplochromini	JN628858	16,583
50	*Dimidiochromis compressiceps*	Haplochromini	JN628856	16,583
51	*Astatotilapia calliptera*	Haplochromini	JN628855	16,578
52	*Pseudotropheus crabro*	Haplochromini	JN628854	16,584
53	*Nimbochromis linni*	Haplochromini	JN628853	16,584
54	*Petrotilapia nigra*	Haplochromini	JN628852	16,583
55	*Diplotaxodon limnothrissa*	Haplochromini	JN628851	16,579
56	*Trematocranus placodon*	Haplochromini	JN628850	16,579
57	*Sarotherodon melanotheron*	Oreochromini	JF894132	16,627
58	*Oreochromis niloticus*	Oreochromini	GU370126	16,625
59	*Tylochromis polylepis*	Tylochromini	AP009509	16,976
60	*Neolamprologus pulcher*	Lamprologini	AP006014	16,587
61	*Tropheus duboisi*	Tropheini	AP006015	16,598
62	*Oreochromis mossambicus*	Oreochromini	AY597335	16,641
63	*Neolamprologus signatus*	Lamprologini	MZ427900	16,583
64	*Pelvicachromis pulcher*	Chromidotilapiini	MZ357707	17,196
65	*Oreochromis tanganicae*	Oreochromini	MK951678	16,632
66	*Hemitilapia oxyrhynchus*	Haplochromini	KX594381	16,583
67	*Dimidiochromis strigatus*	Haplochromini	KX346235	16,582
68	*Oreochromis aureus*	Oreochromini	GU370125	16,628
69	*Mugil cephalus*	Outgroup	AP002930	16,685
