# Supplementary material for: Mitogenomic Characterization of Cameroonian Endemic Coptodon camerunensis (Cichliformes: Cichlidae) and Matrilineal Phylogeny of Old-World Cichlids
Source: Genes (Basel). 2023 Aug 6;14(8):1591. doi: 10.3390/genes14081591 (PMC10454717; doi:10.3390/genes14081591)
Supplement: Supplementary file 1 [file genes-14-01591-s001.zip › Table S2.pdf]

Table S2. Comparison of the intergenic nucleotides of two *Coptodon* species mitogenomes.

Genes	SPECIES		
	Coptodon camerunensis (OQ696044)	*Coptodon zillii* (MW194077)	*Coptodon zillii* (KM658974)
tRNA-Phe (F)	0	0	0
12S rRNA	0	1	0
tRNA-Val (V)	0	0	0
16S rRNA	0	0	0
tRNA-Leu (L2)	0	0	0
ND1	3	3	3
tRNA-Ile (I)	-1	-1	-1
tRNA-Gln (Q)	-1	-1	-1
tRNA-Met (M)	0	0	0
ND2	0	0	0
tRNA-Trp (W)	1	1	1
tRNA-Ala (A)	1	1	0
tRNA-Asn (N)	35	35	33
tRNA-Cys (C)	0	0	0
tRNA-Tyr (Y)	1	1	1
COI	0	0	0
tRNA-Ser (S2)	3	3	3
tRNA-Asp (D)	5	5	5
COII	0	0	0
tRNA-Lys (K)	1	1	1
ATP8	-10	-10	-10
ATP6	0	-1	-1
COIII	0	0	0
tRNA-Gly (G)	0	0	0
ND3	0	0	0
tRNA-Arg (R)	0	0	0
ND4L	-7	-7	-7
ND4	0	0	0
tRNA-His (H)	0	-1	0
tRNA-Ser (S1)	4	4	4
tRNA-Leu (L1)	0	0	0
ND5	-4	-4	-4
ND6	0	0	0
tRNA-Glu (E)	4	4	4
Cyt b	0	0	0
tRNA-Thr (T)	0	0	0
tRNA-Pro (P)	-69	0	0
Control region			
