# Supplementary material for: Mitogenomic Characterization of Cameroonian Endemic Coptodon camerunensis (Cichliformes: Cichlidae) and Matrilineal Phylogeny of Old-World Cichlids
Source: Genes (Basel). 2023 Aug 6;14(8):1591. doi: 10.3390/genes14081591 (PMC10454717; doi:10.3390/genes14081591)
Supplement: Supplementary file 1 [file genes-14-01591-s001.zip › Table S3.pdf]

Table S3. Start and stop codons of all 13 PCGS in two *Coptodon* species mitogenomes.

**PCGs**	*Coptodon camerunensis* (OQ696044)		*Coptodon zillii* (MW194077)		*Coptodon zillii* (KM658974)	
	**START**	**STOP**	**START**	**STOP**	**START**	**STOP**
ND1	ATG	TAA	ATG	TAG	ATG	TAG
ND2	ATG	TA-	ATG	TA-	ATG	TA-
COI	GTG	TAA	GTG	TAA	GTG	TAG
COII	ATG	T--	ATG	T--	ATG	T--
ATP8	ATG	TAA	ATG	TAA	ATG	TAA
ATP6	ATG	TA-	ATG	TA-	ATG	TA-
COIII	ATG	T--	ATG	T--	ATG	T--
ND3	ATG	T--	ATG	T--	ATG	T--
ND4L	ATG	TAA	ATG	TAA	ATG	TAA
ND4	ATG	T--	ATG	T--	ATG	T--
ND5	ATG	TAA	ATG	TAA	ATG	TAA
ND6	ATG	TAG	ATG	TAA	ATG	TAA
Cyt b	ATG	T--	ATG	T--	ATG	T--
